# Expression of Recipient CD47 on Rat Insulinoma Cell Xenografts Prevents Macrophage-Mediated Rejection through SIRPα Inhibitory Signaling in Mice

**DOI:** 10.1371/journal.pone.0058359

**Published:** 2013-03-05

**Authors:** Yoshifumi Teraoka, Kentaro Ide, Hiroshi Morimoto, Hiroyuki Tahara, Hideki Ohdan

**Affiliations:** Department of Surgery, Division of Frontier Medical Science, Programs for Biomedical Research, Graduate School of Biomedical Sciences, Hiroshima University, Hiroshima, Japan; Institut Jacques Monod, France

## Abstract

We have previously proven that the interspecies incompatibility of CD47 is responsible for *in vitro* phagocytosis of xenogeneic cells by host macrophages. Utilizing an *in vivo* model in the present study, we investigated whether genetically engineered expression of mouse CD47 in rat insulinoma cells (INS-1E) could inhibit macrophage-mediated xenograft rejection. INS-1E cells transfected with the pRc/CMV-mouse CD47 vector (mCD47-INS-1E) induced SIRPα-tyrosine phosphorylation in mouse macrophages in vitro, whereas cells transfected with the control vector (cont-INS-1E) did not. When these cells were injected into the peritoneal cavity of streptozotocin-induced diabetic Rag2^−/−^γ chain ^−/−^ mice, which lack T, B, and NK cells, the expression of mouse CD47 on the INS-1E cells markedly reduced the susceptibility of these cells to phagocytosis by macrophages. Moreover, these mice became normoglycemic after receiving mCD47-INS-1E, whereas the mice that received cont-INS-1E failed to achieve normoglycemia. Furthermore, injection of an anti-mouse SIRPα blocking monoclonal antibody into the mouse recipients of mCD47-INS-1E cells prevented achievement of normoglycemia. These results demonstrate that interspecies incompatibility of CD47 significantly contributes to *in vivo* rejection of xenogeneic cells by macrophages. Thus, genetic induction of the expression of recipient CD47 on xenogeneic donor cells could provide inhibitory signals to recipient macrophages via SIPRα; this constitutes a novel approach for preventing macrophage-mediated xenograft rejection.

## Introduction

Xenotransplantation, using organs, tissues, and cells from other species as the transplant source, has the potential to resolve the severe shortage of human donors; however, robust immune responses to xenografts remain a major obstacle to the clinical application of this approach [1], [2]. Extensive genetic disparities between the donor and recipient are thought to contribute significantly to the more vigorous rejection of xenografts than allografts; these disparities, however, remain ill-defined. Vigorous innate immune cell activation can be accounted for by both recognition of xenoantigens by activating receptors, and an incompatibility in inhibitory receptor–ligand interactions [3].

In human xenograft recipients, innate humoral and cellular xenoimmune responses are both predominantly elicited by preformed and induced xenoreactive antibodies (Abs). Among the components of innate immunity, macrophages, which can be activated by prophagocytic signaling pathways through Fcγ receptors, play a significant role in targeting xenogeneic cells that have been opsonized with these Abs. Genetically engineered α-1,3-galactosyltransferase (GalT)-knockout pigs, which no longer express the major xenoantigens Galα1,3Galβ1,4GlcNAc (Gal) carbohydrate residues held promise for conferring protection against such xenoreactive Ab-mediated rejection [4]–[6].

However, we have previously demonstrated that human reticuloendothelial macrophages can phagocytose porcine cells even in the absence of Ab or complement opsonization, and that removing Gal epitopes from porcine cells failed to prevent this phagocytosis [7]. Similarly, other groups have also reported that non-human primate macrophages mediate rapid rejection of porcine pancreatic islets [8], which express little or no Gal antigens [9]. These results suggest that regulation of macrophages in human recipients may be required to achieve successful engraftment of porcine xenografts.

We have recently proven that the interspecies incompatibility of CD47 is responsible for *in vitro* phagocytosis of xenogeneic porcine cells by human macrophages [10]. CD47 is an ubiquitously expressed cell surface protein of the immunoglobulin superfamily that serves as a ligand for signal regulatory protein (SIRP) α, an immune inhibitory receptor on macrophages. CD47 and SIRPα constitute a cell–cell communication system (the CD47-SIRPα system); such interactions play important roles in both hematopoietic and immunological regulation [11]–[14]. In addition, the CD47-SIRPα system has been implicated in negative regulation of phagocytosis by macrophages [15]; specifically, when expressed on the surface of several cell types (i.e., erythrocytes, platelets, or leukocytes), CD47 can protect against phagocytosis by macrophages by binding to SIRPα [15]. Moreover, CD47 inhibits both Fcγ and complement receptor-mediated phagocytosis through its SIRPα receptors [16].

We have previously verified that porcine CD47 does not induce tyrosine phosphorylation of SIRPα in human macrophages, and that manipulation of porcine cells for expression of human CD47 markedly reduces the susceptibility of these cells to phagocytosis by human macrophages *in vitro* [10]. These results indicated that genetic induction of the expression of recipient-type CD47 on xenogeneic donor cells could provide inhibitory signaling to SIPRα on host macrophages, suggesting a novel approach for preventing macrophage-mediated xenograft rejection.

Here, we have now investigated this postulate in a rat-to-mouse *in vivo* model, in which the interspecies incompatibility of CD47 (85% amino acid sequence homology between these species [17]) would normally cause active phagocytosis of rat cells by mouse macrophages.

## Materials and Methods

### Antibodies

An anti-SIRPα Ab (P84) was used to block the macrophage inhibitory receptor, SIRPα [18]. Biotin-conjugated rat anti-mouse CD11b (M1/70; BD Pharmingen, San Diego, CA, USA), purified anti-mouse CD47 Ab (miap301; BD Pharmingen), and APC Streptavidin (BD Pharmingen) were used for FACS analysis via immunofluorescence using a FACSCalibur® (BD Biosciences, Franklin Lakes, NJ, USA). In FACS analyses, nonspecific binding of labeled mAbs was blocked with rat anti–mouse FCγR mAb, 2.4G2. Rabbit polyclonal Ab against SIRPα (Abcam, La Jolla, CA, USA), purified rat anti-mouse CD172a (P84) Ab (BD Pharmingen), purified rabbit polyclonal anti-phosphotyrosine Ab (BD Transduction Laboratories, Lexington, KY, USA), and horse radish peroxidase (HRP)-conjugated rabbit secondary Ab (Amersham Biosciences Co., Piscataway, NJ, USA) were used for immunoprecipitation and western blot analysis.

### Cell Cultures

All cells were maintained at 37°C under a humidified atmosphere of 5% CO2 in air. A rat insulinoma cell line (INS-1E) [19] was kindly provided by Dr. Claes B. Wollheim (University of Geneva, Switzerland). Cells were cultured in RPMI1640 containing 10% FCS with 5 µM 2-mercaptoethanol (2-ME; Katayama, Osaka, Japan), 10% HEPES buffer (Gibco, NY, USA), and 100 IU/mL penicillin-100 µg/mL streptomycin (Gibco). P84 hybridoma cells producing an anti-SIRPα Ab were kindly provided by T. Matozaki (Gunma University, Gunma, Japan). The mouse macrophages were cultured in DMEM containing 10% FCS with 5 µM 2-ME, 10% HEPES buffer, and 100 IU/mL penicillin-100 µg/mL streptomycin.

### Animals

Rag2^−/−^ γ chain ^−/−^ mice were purchased from Taconic (One Hudson City Centre Hudson, NY, USA). All animal protocols described in this study were performed in accordance with the Guide for the Care and Use of Laboratory Animals and the local committee for animal experiments, and the experimental protocol was approved by the Ethics Review Committee for Animal Experimentation of the Graduate School of Biomedical Sciences, Hiroshima University.

### Mouse Macrophage Preparation

To prepare peritoneal macrophages, peritoneal cells were harvested from B6 mice after intraperitoneal injection of PBS, plated in a Gelatin Cellware 75-cm2 Vented Flask (BD Biosciences) and cultured at 37°C for 2 h. Macrophages were used after non-adherent cells were washed off.

### T Cell Isolation

T cells were negatively isolated from wild type B6 splenocytes using a cocktail of biotin-conjugated monoclonal antibodies against CD11b, CD11c, CD19, CD45R (B220), CD49b (DX5), CD105, anti-MHC class II, and Ter-119 and anti-biotin antibody-coated magnetic beads (Miltenyi Biotec, Bergisch Gladbach, Germany) according to the manufacturer’s instructions. The purity of T cells was ≥95% (data not shown), as determined by CD3e surface staining using FACS analysis.

### Immunoprecipitation and Immunoblotting

Peritoneal macrophages (2×106) were incubated for 16 h before experiments and rinsed once with PBS. Then, 2×107 mouse or rat RBCs or INS-1E cells were added to the macrophage cultures, and incubated at 37°C for 30 min. The cells were lysed in 0.3 mL of lysis buffer [1% NP-40, 1 mM PMSF, 50 mM β-glycophosphate, 20 mM NaF, 0.5 µg/mL leupeptin, 0.5 µg/mL aprotinin, and 2 mM sodium pervanadate] by rotation on ice for 15 min.

For immunoprecipitation, the lysates were mixed with rat anti-mouse SIRPα Ab (P84) and 50% slurry of protein G–sepharose beads (Sigma-Aldrich, St. Louis, MO, USA) by rotation at 4°C for 8 h. Precipitated proteins were separated by 8% SDS-PAGE and transferred to nitrocellulose membrane. Thereafter, polyclonal rabbit anti-phosphotyrosine Ab (BD Pharmingen) and HRP-linked anti-rabbit IgG Ab (Amersham Biosciences Co.) were used as primary and secondary Abs, respectively, in western blot analysis. Alternatively, the membrane was stained with rabbit polyclonal Ab against SIRPα (Abcam), followed by HRP-linked anti-rabbit IgG Ab (Amersham Biosciences Co.).

### Mouse CD47 cDNA Plasmid Construction and Transfection

The entire coding region of the CD47 cDNA was PCR-amplified from reverse-transcribed mouse lymphocyte cDNA with primers (sense) 5′-GCGAAGTGACAGAGTTATCC-3′ and (antisense) 5′-TGGCTCACATGCCATGATGC-3′. The amplified PCR product was digested with *Eco*RI/*Not*I and cloned into the pRc/CMV vector (kindly provided by Dr. Tadashi Furusawa, National Institute of Animal Research Industry, Japan), which had been predigested with the same restriction endonucleases. Rat insulinoma cells (INS-1E) were transfected with either pRc/CMV-mouse CD47 or the empty plasmid, using Lipofectamine 2000 (Invitrogen, Carlsbad, CA, USA). Five hours after the transfection, cells were selected with G418 (Sigma-Aldrich; 800 µg/mL) for 1 week to generate stable cell lines and analyzed for expression of mouse CD47 by FACS analyses.

### Diabetic Mice Generation

Rag2^−/−^ γ chain ^−/−^ mice were rendered diabetic through a single i.p. administration of 200 mg/kg streptozotocin (Sigma-Aldrich) at 6 days prior to injection of rat INS-1E cells. Diabetic mice with non-fasting blood glucose levels of >400 mg/dL on the day of transplantation were used as the recipients. The blood glucose levels were monitored with a blood glucose test meter (Medisafemini GR-102; Terumo, Somerset, NJ, USA). In the absence of INS-1E cell transplantation, diabetes persisted in all diabetic mice (blood glucose level: 0.350 mg/dL), and no spontaneous reversal of diabetes was observed for at least the next 3 months.

### *In vivo* Phagocytic Assay

Target cells were stained with the fluorescent dye 5/6-CFSE (Molecular Probes, Eugene, OR, USA) according to the manufacturer’s protocol. Either CFSE-labeled mCD47-INS-1E cells (10×10^6^) or cont-INS-1E cells (10×10^6^) were injected into the peritoneal cavity of streptozotocin-induced diabetic Rag2^−/−^ γ chain ^−/−^ mice. After 6 h, the recipient intraperitoneal cells were harvested and the macrophages that phagocytosed the target cells could be identified by FACS analysis, based on CFSE labeling.

#### Statistical analysis

Significant differences between groups were determined using Student’s t-test. A p-value of <0.05 was considered statistically significant.

## Results

### Rat CD47 does not Induce Tyrosine Phosphorylation of SIRPα in Mouse Macrophages

In the CD47-SIRP system, the interaction between SIRP, on macrophages, and CD47, on target cells, inhibits phagocytosis of the target cells by promoting phosphorylation of tyrosine in the cytoplasmic domain, and recruitment of Src homology 2 domain-containing protein tyrosine phosphatase-1, which is the major regulator of phagocytic responses [20].

**Figure 1:**
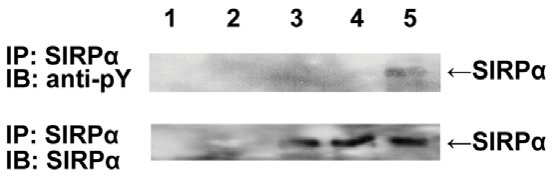
Tyrosine phosphorylation of SIRPα in mouse macrophages was induced by incubation with mouse red blood cells (RBCs), but not with rat RBCs. Differentiated mouse macrophages were incubated with mouse or rat RBCs at 37°C for 30 min. The cells were lysed, and the lysates were mixed with mouse anti-mouse SIRPα antibodies and 50% slurry of protein G-sepharose beads by rotation at 4°C for 8 hrs. Precipitated proteins were separated by 8% SDS-PAGE, followed by blotting to a nitrocellulose membrane. Rabbit immunoaffinity-purified anti-phosphotyrosine IgG and goat anti-rabbit HRP-conjugated IgG were used as primary and secondary antibodies, respectively. Rat RBCs alone (lane 1), mouse RBCs alone (lane 2), mouse macrophages incubated in medium alone (lane 3), or mouse macrophages incubated with rat (lane 4) or mouse (lane 5) RBCs are shown. Immunoblotting with anti-mouse SIRPα was used as loading control. IP, immunoprecipitation; IB, immunoblotting; anti-pY, anti-phosphotyrosine.

To determine whether rat CD47 can interact with mouse SIRPα, we assessed tyrosine phosphorylation of SIRPα in mouse macrophages after contact with either rat or mouse RBCs. Western blotting revealed that incubation of mouse peritoneal cavity macrophages with mouse RBCs resulted in SIRPα tyrosine phosphorylation, as expected (Fig. 1). However, after incubation with rat RBCs, this tyrosine phosphorylation was not induced in mouse macrophages above the level in control macrophages, which had been incubated with medium alone, indicating that rat CD47 fails to induce SIRPα tyrosine phosphorylation in mouse macrophages.

### Mouse CD47 Expression on Rat Cells Markedly Reduces the Susceptibility to Phagocytosis by Mouse Macrophages

**Figure 2:**
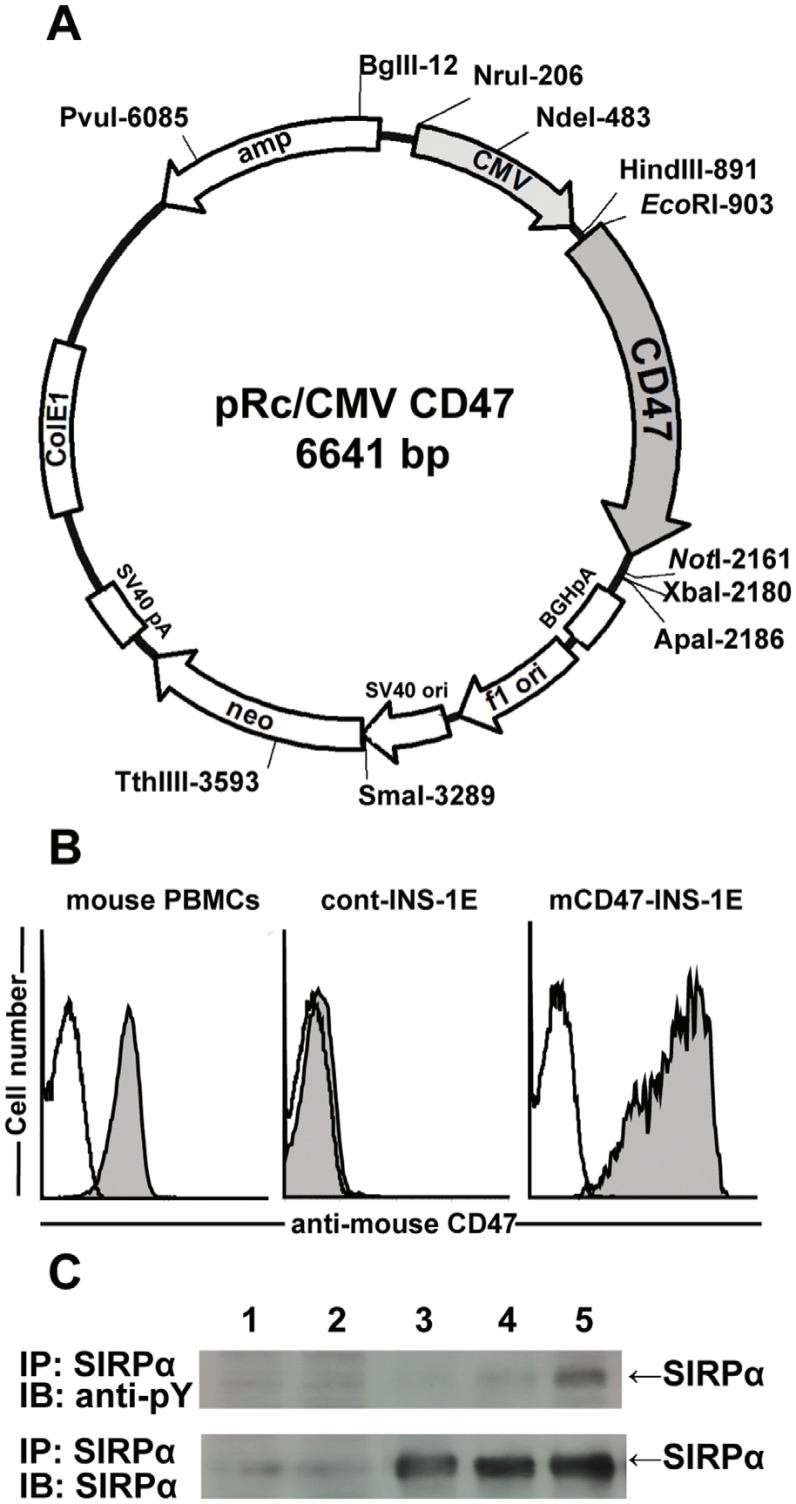
Generation of mouse CD47-expressing rat cell line. (A) Structure of pRc/CMV-mouse CD47. The entire coding region of the mouse CD47 cDNA was PCR-amplified. The amplified PCR product was digested and full-length mouse CD47 cDNA was inserted into the expression vector pRc/CMV. (B) Expression of mouse CD47 on a transfected rat insulinoma cell (INS-1E) was confirmed by FACS analysis. Representative histograms obtained by FACS analysis for mouse PBMCs, pRc/CMV-transfected rat INS-1E cells (cont-INS-1E), and pRc/CMV-mouse CD47-transfected rat INS-1E cells (mCD47-INS-1E) are shown. Open and filled histograms represent staining with isotype control and with anti-mouse CD47 mAb, respectively. (C) Tyrosine phosphorylation of SIRPα in mouse macrophages was induced by incubation with pRc/CMV-mouse CD47-transfected rat INS-1E cells (mCD47-INS-1E), but not with control vector-transfected rat INS-1E cells (cont-INS-1E). Differentiated mouse macrophages were incubated with mCD47-INS-1E or cont-INS-1E at 37°C for 30 min. The cells were lysed, and the lysates were mixed with mouse anti-mouse SIRPα antibodies and 50% slurry of protein G-sepharose beads by rotation at 4°C for 8 hrs. Precipitated proteins were separated by 8% SDS-PAGE, followed by blotting to a nitrocellulose membrane. Rabbit immunoaffinity-purified anti-phosphotyrosine IgG and goat anti-rabbit HRP-conjugated IgG were used as primary and secondary antibodies, respectively. Mouse CD47-transfected INS-1E (mCD47-INS-1E) alone (lane 2), mouse macrophages incubated in medium alone (lane 3) or mouse macrophages incubated with cont-INS-1E (lane 4) or mCD47-INS-1E (lane 5) are shown. Immunoblotting with anti-mouse SIRPα was used as loading control. IP, immunoprecipitation; IB, immunoblotting; anti-pY, anti-phosphotyrosine.

To determine whether expression of mouse CD47 on rat cells could efficiently prevent their phagocytosis by mouse macrophages, we generated rat insulinoma cell lines that express mouse CD47 by transfecting rat cells with a mouse CD47-expressing plasmid, pRc/CMV-mouse CD47 (Fig. 2A). Mouse CD47 expression on the transfected INS-1E cells was confirmed by FACS analysis (Fig. 2B). The expression level of CD47 on the pRc/CMV-mouse CD47 vector-transfected INS-1E cells (mCD47-INS-1E) was higher than that on mouse PBMCs, whereas the control vector-transfected INS-1E (cont-INS-1E) cells tested negative for mouse CD47. We have confirmed constant expression of mouse CD47 on mCD47-INS-1E cells in diabetic Rag2^−/−^ γ chain^−/−^ mice without further G418-selection at least until 8 days after the inoculation. Western blotting revealed that incubation of mouse macrophages with mCD47-INS-1E resulted in significant tyrosine phosphorylation of SIRPα, indicating that mCD47-INS-1E cells functionally interact with mouse SIRPα (Fig. 2C). Except for functional mouse CD47 expression, mCD47-INS-1E cells were comparable to cont-INS-1E cells with respect to their morphology, proliferation rates, and insulin producing activity (data not shown).

**Figure 3:**
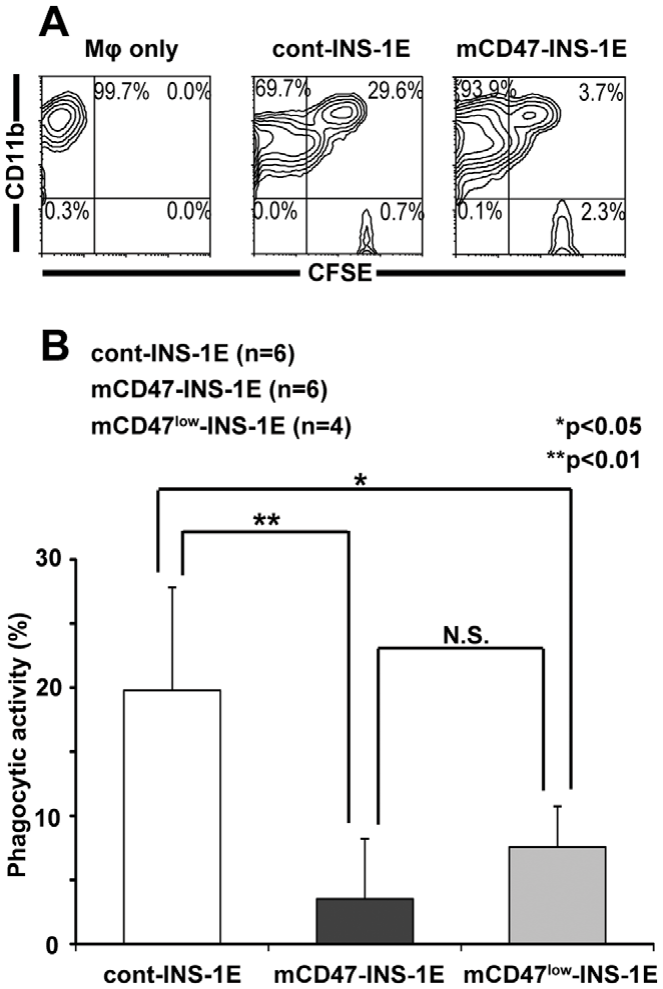
Mouse CD47-expressing rat INS-1E cells attenuate phagocytosis by mouse macrophages. (A) CFSE-labeled pRc/CMV-mouse CD47-transfected rat INS-1E cells (mCD47-INS-1E) and control vector-transfected rat INS-1E cells (cont-INS-1E) were injected into peritoneal cavity of streptozotocin-induced diabetic Rag2^−/−^ γ chain ^−/−^ mice. After 6 h, the intraperitoneal cells from the recipient mice were harvested. Mouse macrophages counterstained with allophycocyanin-conjugated anti-mouse CD11b and phagocytosis of CFSE-labeled targets were measured by FACS analysis. Representative FACS profiles are shown. Regions representing non-phagocytosing macrophages are shown in the upper left quadrants, regions representing phagocytosing macrophages are shown in the upper right quadrants, and regions representing residual targets are shown in the lower right quadrants. (B) Phagocytic activity was calculated by the following formula: phagocytic activity = (percentage of engulfing macrophages/percentage of total harvested macrophages) ×100. Data are given as the means ± SD.

The phagocytic activities of mouse macrophages toward both INS-1E cell lines were evaluated by *in vivo* assays. CFSE-labeled mCD47-INS-1E or cont-INS-1E cells were injected into the peritoneal cavity of streptozotocin-induced diabetic Rag2^−/−^ γ chain ^−/−^ mice, which lack T, B, and NK cells. The recipient intraperitoneal cells were harvested after 6 h, and macrophages that phagocytosed the target cells were then detected as CD11b- and CFSE-double–positive cells, using FACS analysis (Fig. 3A). The proportion of CFSE^+^ cells among all the CD11b^+^ cells was significantly lower in cells obtained from mCD47-INS-1E recipients than from cont-INS-1E recipients. This result indicated that mouse CD47 expression on rat cells markedly reduced the susceptibility of these cells to phagocytosis by mouse macrophages (Fig. 3B).

To investigate whether these protections were due to CD47 overexpression or to the species-specific effect of mouse CD47, another line of rat insulinoma cells (mCD47^low^-INS-1E) labeled with CFSE, which expressed lower levels of mouse CD47 (MFI 330.67) than the original mCD47-INS-1E cells (MFI 506.88), were injected into the peritoneal cavity of streptozotocin-induced diabetic Rag2^−/−^ γ chain^−/−^ mice. Intraperitoneal cells of the recipients were harvested after 6 h, and macrophages that phagocytosed the target cells were detected using FACS analysis. The proportion of CFSE^+^ cells among all CD11b^+^ cells was significantly lower in cells obtained from mCD47^low^-INS-1E recipients than in those from cont-INS-1E recipients. However, no significant difference was observed in phagocytic activity between mCD47-INS-1E recipients and mCD47^low^-INS-1E recipients. This result indicated that, in this model, the protection was not simply due to overexpression of mouse CD47 (Fig. 3B).

### Diabetic Rag2^−/−^ γ chain ^−/−^ Mice became Normoglycemic after Receiving mCD47-INS-1E

**Figure 4:**
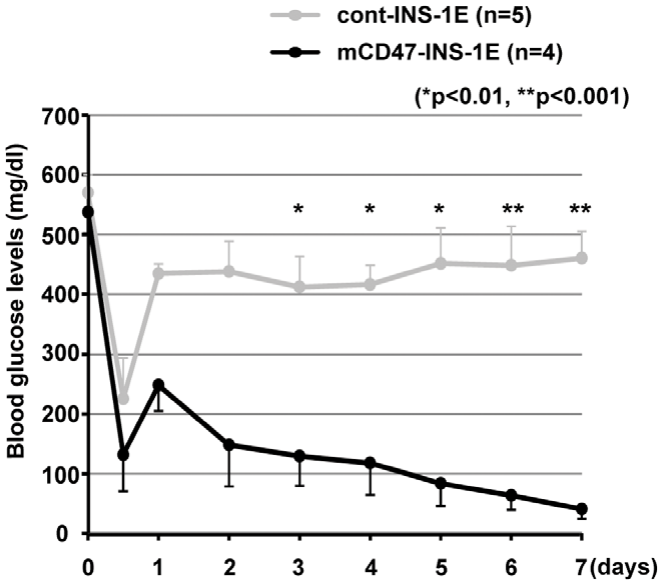
Diabetic Rag2^−/−^ γ chain ^−/−^ mice became normoglycemic after receiving mCD47-INS-1E. Either mCD47-INS-1E or cont-INS-1E cells were injected into the peritoneal cavity of Rag2^−/−^ γ chain ^−/−^ mice with streptozotocin-induced diabetes. Blood glucose levels were monitored for 7 days. Data are presented as the means ± SD.

Next, mCD47-INS-1E or cont-INS-1E cells were injected into the peritoneal cavity of Rag2^−/−^ γ chain ^−/−^ mice with streptozotocin-induced diabetes; the blood glucose levels of these mice were monitored for 7 days. Diabetic Rag2^−/−^ γ chain ^−/−^ mice became normoglycemic after receiving mCD47-INS-1E. In contrast, the mice that received cont-INS-1E failed to achieve normoglycemia (Fig. 4). Thus, the in vivo transplant model proved that genetically engineered expression of mouse CD47 in rat insulinoma cells could inhibit macrophage-mediated xenograft rejection.

### CD47-SIRPα Signaling Blockade Prevents the Effects of Mouse CD47 on Macrophage-mediated Xenograft Rejection

**Figure 5:**
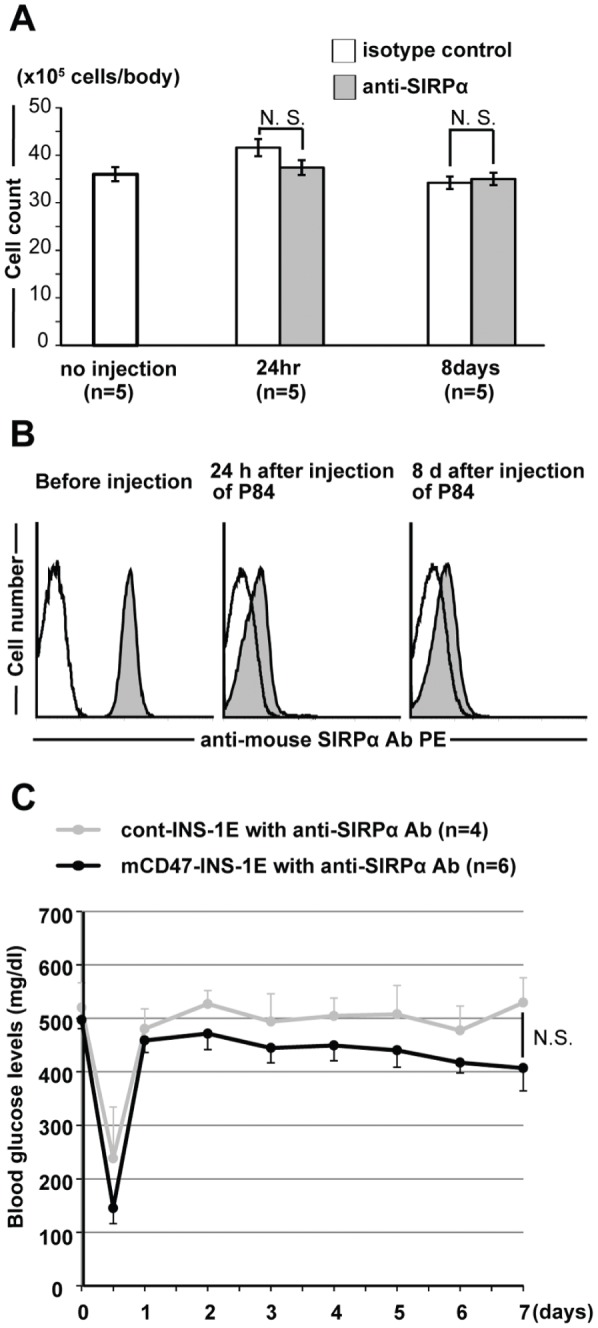
Inhibition of CD47-SIRPα signaling prevents the effect of genetic induction of recipient CD47 in xenografts. (A) Either P84 or control antibody was injected into the peritoneal cavity of diabetic Rag2^−/−^ γ chain^−/−^ mice. After injection, intraperitoneal cells from recipient mice were harvested and SIRPα+ peritoneal cells were counted. (B) Anti-mouse SIRPα mAb (P84) was injected into the peritoneal cavity of Rag2−/− γ chain −/− mice. Expression of mouse SIRPα on mouse peritoneal macrophages was confirmed by FACS analysis. Open and filled histograms represent staining with isotype control and with anti-mouse SIRPα mAb, respectively. (C) Twenty-four hours after the injection of anti-mouse SIRPα mAb (P84), either mCD47-INS-1E or cont-INS-1E cells were injected into the peritoneal cavity of the diabetic Rag2^−/−^ γ chain ^−/−^ mice. Blood glucose levels were monitored for 7 days. Data are given as the means ± SD. N.S.: not significant.

We further explored the practical contribution of CD47-SIRPα signaling to the successful engraftment of mCD47-INS-1E xenografts in the diabetic Rag2^−/−^ γ chain ^−/−^ mice by using the anti-mouse SIRPα Ab (P84) to block this signaling. Mouse SIRPα, on the peritoneal macrophages of Rag2−/− γ chain −/− mice, had not been decreased and had been capped after the intraperitoneal injection of P84 for at least 8 days (Fig. 5A and B). Twenty-four hours after P84 injection, either mCD47-INS-1E or cont-INS-1E cells were injected into the peritoneal cavity of the diabetic Rag2^−/−^ γ chain ^−/−^ mice. Irrespective of which cell line recipients received, mice failed to achieve normoglycemia (Fig. 5C), indicating the essential role of CD47-SIRPα signaling in overcoming macrophage-mediated rejection of xenografts.

### CD47-SIRPα Signaling Blockade does not Induce Phagocytosis of Congenic Cells

**Figure 6:**
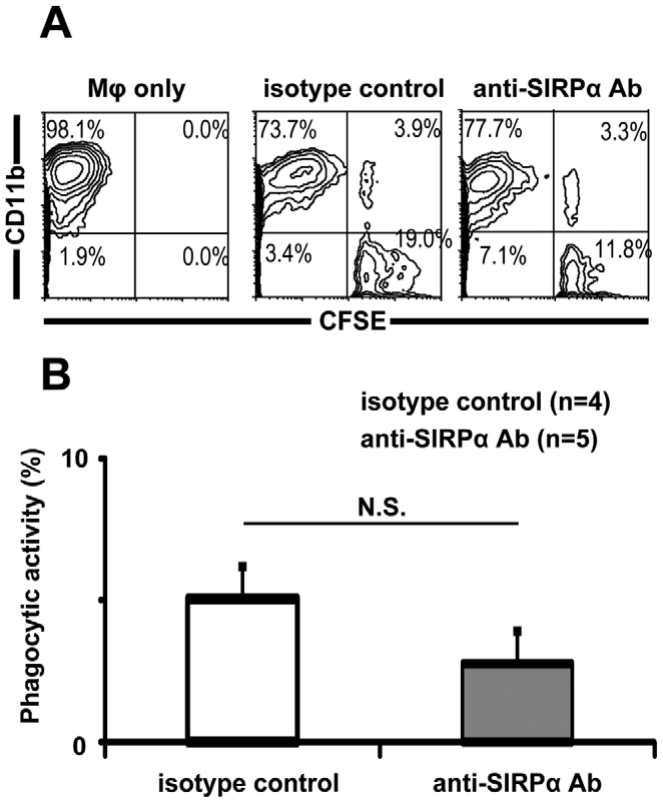
CD47-SIRPα signaling blockade does not induce phagocytosis of congenic cells. (A) Twenty-four hours after injection of either P84 or control antibody, CFSE-labeled congenic T cells were injected into the peritoneal cavity of mice. After 6 h, the intraperitoneal cells from recipient mice were harvested. Mouse macrophages counterstained with allophycocyanin-conjugated anti-mouse CD11b and phagocytosis of CFSE-labeled congenic T cells were determined by FACS analysis. Representative FACS profiles are shown. Regions representing non-phagocytosing macrophages are shown in the upper left quadrants, regions representing phagocytosing macrophages are shown in the upper right quadrants, and regions representing residual congenic T cells are shown in the lower right quadrants. (B) Phagocytic activity was calculated by using the following formula: phagocytic activity = (percentage of engulfing macrophages/percentage of total harvested macrophages)×100. Data are presented as means ± SD.

We further examined whether injection of P84 into the peritoneal cavity of diabetic Rag2^−/−^ γ chain^−/−^ mice results in phagocytosis by mouse cells. Twenty-four hours after injection of either P84 or control antibody, CFSE-labeled congenic T cells were injected into the peritoneal cavity of mice. After 6 h, intraperitoneal cells of the recipients were harvested and mouse macrophages that phagocytosed congenic T cells were then detected as CD11b- and CFSE-double–positive cells using FACS analysis. As shown in Fig. 6, no significant difference was observed in phagocytic activity between the 2 groups. This result indicated that CD47-SIRPα signaling blockade does not induce phagocytosis of congenic cells.

## Discussion

In the present study, genetic induction of the expression of mouse CD47 on rat insulin-producing cells could deliver inhibitory signaling to SIPRα on mouse peritoneal macrophages, preventing rejection of the rat cells during observation periods. It has previously been reported that CD47-SIRPα interactions exhibit limited cross-species reactivity probably because of species-specific posttranslational modifications of CD47 such as glycosylation, i.e. CD47 on pig but not on mouse, cow, or rat RBCs binds the recombinant extracellular dommain of human SIRPα1 [21]. It has been also demonstrated that pig CD47 does not interact with mouse SIRPα [22]. Consistently, the phagocytic synapse at cell contacts has been proven to involve a basal level of actin-driven phagocytosis that is made more efficient by phospho-activated myosin in the absence of species-specific CD47 signaling [23]. Recently, we have verified that pig CD47 also does not interact with human SIRPα, and, importantly, that genetic manipulation of porcine cells for expression of human CD47 markedly reduces the susceptibility of these cells to phagocytosis by human macrophages *in vitro* [10].

Other groups have also shown that functional species-specific CD47/SIRPα interaction is required for generating improved models of mouse/human chimeras: mouse CD47-expression in transplanted human hematopoietic cells is required for optimal human T- and natural killer-cell homeostasis in mice [24]. Furthermore, the introduction of mouse CD47 into primary human hepatocytes confers a positive selective advantage upon engraftment into the mouse liver *in vivo* [25]. Currently available data from in vivo experiments assessing xenograft survival indicates that CD47 provides a potential molecular target for inhibiting macrophage-mediated rejection of xenogeneic cells. Hence, this suggests the necessity of establishing human CD47-transgenic pigs as clinically applicable donors of xenografts.

It is well known that innate immune responses mediated by monocytes/macrophages can drive and shape the process of adaptive immunity. Phagocytic activities of macrophages form a first line of defense against invading infectious microbes, and these macrophages can present antigens derived from such phagocytosed foreign pathogens to T cells. It is likely that these mechanisms also take place in xenotransplantation from phylogenetically distant species. Therefore, specific elimination of phagocytic activity of host macrophages toward xenogeneic cells by genetically inducing host-type CD47 expression may also attenuate subsequent T cell immune responses against xenoantigens, while maintaining normal responses against other pathogens.

It has also been reported that a similar CD47-SIRP system negatively regulates the functions of both T cells and APCs in humans [14]. In contrast, it has been demonstrated that the interaction between CD47 on APCs and SIRPγ (also known as SIRPβ2) on T cells promotes the proliferation of antigen-specific T cells and co-stimulates T cell activation [26]. These observations raise a question as to whether interspecies incompatibility of CD47 affects CD4+ T cell-mediated responses to xenoantigens positively or negatively. In our previous study, recombinant human CD47-Fc fusion protein (which contains the extracellular domain of human CD47 fused to the Fc portion of human immunoglobulin) significantly reduced the indirect response of human CD4+ T cells to porcine antigens, but did not affect the direct response of these cells in in vitro pig-to-human mixed lymphocyte reaction assays [27]. Inhibition of the phagocytic activity of human APCs toward porcine cells by interaction between the human CD47-Fc fusion protein and the corresponding ligand, probably SIRPα, might might attenuate subsequent CD4+ T cell immune responses against porcine antigens. Taking into consideration that SIRPγ binds CD47 with a lower affinity (KD: about 23 µM) than SIRPα (KD: about 2 µM) [28], the interaction between human CD47-Fc and SIRPγ on human CD4+ T cells conferring direct xenospecificity may might not affect CD4+ T cell immune responses. Unlike this in vitro system, however, if human CD47 molecules are highly expressed on porcine APCs by genetic manipulations, there will be a risk that CD4+ T cell-mediated responses to xenoantigens are promoted. These possibilities should be addressed in further studies employing an immunocompetent animal model enabling long-term observation. In that particular case, the model utilizing rat inslinoma in mice would not be suitable, since the mouse recipients of mCD47-INS-1E cells in this study eventually died from hypoglycemia due to growth of the inocula. Further studies are needed for long-term observation employing a rat CD47-transfected normal mouse islet model.

It has been reported that the ability of glucocorticoids to promote macrophage phagocytosis of CD47-deficient targets could, in part, be mediated by an upregulation of expression of LDL receptor-related protein-1 (LRP1/CD91/α2-macroglobulin receptor) macrophages [29]. Since glucocorticoids are indispensable in immunosuppressive therapy after xenogeneic or even allogeneic cellular/organ transplantation, glucocorticoid-treated macrophages may enhance phagocytosis of xenogeneic cells. Therefore, genetic manipulation of xenogeneic cells for host-type CD47 expression would be particularly useful to reduce the likelihood of phagocytosis by macrophages.

It has been recently demonstrated that CD47 is a molecule commonly expressed on neoplastic cells. Its function to block phagocytosis is known, and blockade of this function leads to tumor cell phagocytosis and elimination [30], [31]. Consistently, in a separate experiment, we also observed that insulinoma cell function in a syngeneic model depends on SIRPα-mediated inhibition of macrophages through engagement with its ligand CD47, i.e., treatment with anti-SIRPα Abs enhanced macrophage-mediated elimination of mouse insulinoma cells in diabetic Rag2^−/−^ γ chain^−/−^ mice (data not shown). It remains to be elucidated whether the observations made in neoplastic cells in this study apply to normal xenografts. However, together with our previous in vitro finding that genetic induction of human CD47 on porcine non-neoplastic lymphoblastoid cells radically reduced the susceptibility of those cells to phagocytosis by human macrophages, our results of the present study may lead to the development of approaches for attenuating macrophage-mediated xenograft rejection by genetic manipulation of porcine cells for human CD47 expression.

In conclusion, we have here demonstrated that interspecies incompatibility of CD47 significantly contributes to *in vivo* rejection of xenogeneic cells by macrophages. Our results imply that genetic induction of recipient CD47 on xenogeneic donor cells could provide inhibitory signals to recipient macrophages via SIPRα; this constitutes a novel approach to prevent macrophage-mediated xenograft rejection.

## References

[1] Cooper DK, Gollackner B, Sachs DH (2002) Will the pig solve the transplantation backlog? Annual review of medicine 53: 133–147. 10.1146/annurev.med.53.082901.103900 11818467

[2] Yang YG, Sykes M (2007) Xenotransplantation: current status and a perspective on the future. Nature reviews. Immunology 7: 519–531. 17571072 10.1038/nri2099

[3] Simon AR, Warrens AN, Sykes M (1999) Efficacy of adhesive interactions in pig-to-human xenotransplantation. Immunol Today 20: 323–330. 10379051 10.1016/s0167-5699(99)01485-1

[4] Phelps CJ, Koike C, Vaught TD, Boone J, Wells KD, et al (2003) Production of alpha 1,3-galactosyltransferase-deficient pigs. Science 299: 411–414. 12493821 10.1126/science.1078942 PMC3154759

[5] Kuwaki K, Tseng YL, Dor FJ, Shimizu A, Houser SL, et al (2005) Heart transplantation in baboons using alpha1,3-galactosyltransferase gene-knockout pigs as donors: initial experience. Nature medicine 11: 29–31. 10.1038/nm1171 15619628

[6] Yamada K, Yazawa K, Shimizu A, Iwanaga T, Hisashi Y, et al (2005) Marked prolongation of porcine renal xenograft survival in baboons through the use of alpha1,3-galactosyltransferase gene-knockout donors and the cotransplantation of vascularized thymic tissue. Nature medicine 11: 32–34. 10.1038/nm1172 15619627

[7] Ide K, Ohdan H, Kobayashi T, Hara H, Ishiyama K, et al (2005) Antibody- and complement-independent phagocytotic and cytolytic activities of human macrophages toward porcine cells. Xenotransplantation 12: 181–188. 15807768 10.1111/j.1399-3089.2005.00222.x

[8] Wu G, Korsgren O, Zhang J, Song Z, van Rooijen N, et al (2000) Pig islet xenograft rejection is markedly delayed in macrophage-depleted mice: a study in streptozotocin diabetic animals. Xenotransplantation 7: 214–220. 11021667 10.1034/j.1399-3089.2000.00071.x

[9] McKenzie IF, Xing PX, Vaughan HA, Prenzoska J, Dabkowski PL, et al (1994) Distribution of the major xenoantigen (gal (alpha 1–3)gal) for pig to human xenografts. Transplant immunology 2: 81–86. 7953322 10.1016/0966-3274(94)90032-9

[10] Ide K, Wang H, Tahara H, Liu J, Wang X, et al (2007) Role for CD47-SIRPalpha signaling in xenograft rejection by macrophages. Proceedings of the National Academy of Sciences of the United States of America 104: 5062–5066. 17360380 10.1073/pnas.0609661104 PMC1829264

[11] Liu Y, Buhring HJ, Zen K, Burst SL, Schnell FJ, Williams IR, et al (2002) Signal regulatory protein (SIRPalpha), a cellular ligand for CD47, regulates neutrophil transmigration. The Journal of biological chemistry 277: 10028–10036. 11792697 10.1074/jbc.M109720200

[12] Motegi S, Okazawa H, Ohnishi H, Sato R, Kaneko Y, et al (2003) Role of the CD47-SHPS-1 system in regulation of cell migration. The EMBO journal 22: 2634–2644. 12773380 10.1093/emboj/cdg278 PMC156773

[13] Yoshida H, Tomiyama Y, Oritani K, Murayama Y, Ishikawa J, et al (2002) Interaction between Src homology 2 domain bearing protein tyrosine phosphatase substrate-1 and CD47 mediates the adhesion of human B lymphocytes to nonactivated endothelial cells. Journal of immunology 168: 3213–3220. 10.4049/jimmunol.168.7.3213 11907074

[14] Latour S, Tanaka H, Demeure C, Mateo V, Rubio M, et al (2001) Bidirectional negative regulation of human T and dendritic cells by CD47 and its cognate receptor signal-regulator protein-alpha: down-regulation of IL-12 responsiveness and inhibition of dendritic cell activation. Journal of immunology 167: 2547–2554. 10.4049/jimmunol.167.5.2547 11509594

[15] Oldenborg PA, Zheleznyak A, Fang YF, Lagenaur CF, Gresham HD, et al (2000) Role of CD47 as a marker of self on red blood cells. Science 288: 2051–2054. 10856220 10.1126/science.288.5473.2051

[16] Oldenborg PA, Gresham HD, Lindberg FP (2001) CD47-signal regulatory protein alpha (SIRPalpha) regulates Fcgamma and complement receptor-mediated phagocytosis. The Journal of experimental medicine 193: 855–862. 11283158 10.1084/jem.193.7.855 PMC2193364

[17] Shahein YE, de Andres DF, Perez de la Lastra JM (2002) Molecular cloning and functional characterization of the pig homologue of integrin-associated protein (IAP/CD47). Immunology 106: 564–576. 12153520 10.1046/j.1365-2567.2002.01465.x PMC1782751

[18] Jiang P, Lagenaur CF, Narayanan V (1999) Integrin-associated protein is a ligand for the P84 neural adhesion molecule. The Journal of biological chemistry 274: 559–562. 9872987 10.1074/jbc.274.2.559

[19] Merglen A, Theander S, Rubi B, Chaffard G, Wollheim CB, et al (2004) Glucose sensitivity and metabolism-secretion coupling studied during two-year continuous culture in INS-1E insulinoma cells. Endocrinology 145: 667–678. 14592952 10.1210/en.2003-1099

[20] Veillette A, Latour S, Davidson D (2002) Negative regulation of immunoreceptor signaling. Annual review of immunology 20: 669–707. 10.1146/annurev.immunol.20.081501.130710 11861615

[21] Subramanian S, Parthasarathy R, Sen S, Boder ET, Discher DE (2006) Species- and cell type-specific interactions between CD47 and human SIRPalpha. Blood 107: 2548–2556. 16291597 10.1182/blood-2005-04-1463 PMC1895743

[22] Wang H, VerHalen J, Madariaga ML, Xiang S, Wang S, et al (2007) Attenuation of phagocytosis of xenogeneic cells by manipulating CD47. Blood 109: 836–842. 17008545 10.1182/blood-2006-04-019794 PMC1785095

[23] Tsai RK, Discher DE (2008) Inhibition of “self” engulfment through deactivation of myosin-II at the phagocytic synapse between human cells. J Cell Biol 180: 989–1003. 18332220 10.1083/jcb.200708043 PMC2265407

[24] Legrand N, Huntington ND, Nagasawa M, Bakker AQ, Schotte R, et al (2011) Functional CD47/signal regulatory protein alpha (SIRP(alpha)) interaction is required for optimal human T- and natural killer- (NK) cell homeostasis in vivo. Proc Natl Acad Sci U S A 108: 13224–13229. 21788504 10.1073/pnas.1101398108 PMC3156191

[25] Waern JM, Yuan Q, Rudrich U, Becker PD, Schulze K, et al (2012) Ectopic expression of murine CD47 minimizes macrophage rejection of human hepatocyte xenografts in immunodeficient mice. Hepatology 56: 1479–1488. 22535707 10.1002/hep.25816

[26] Piccio L, Vermi W, Boles KS, Fuchs A, Strader CA, et al (2005) Adhesion of human T cells to antigen-presenting cells through SIRPbeta2-CD47 interaction costimulates T-cell proliferation. Blood 105: 2421–2427. 15383453 10.1182/blood-2004-07-2823

[27] Tahara H, Ide K, Basnet N, Tanaka Y, Ohdan H (2010) Determination of the precursor frequency and the reaction intensity of xenoreactive human T lymphocytes. Xenotransplantation 17: 188–196. 20636539 10.1111/j.1399-3089.2010.00575.x

[28] Brooke G, Holbrook JD, Brown MH, Barclay AN (2004) Human lymphocytes interact directly with CD47 through a novel member of the signal regulatory protein (SIRP) family. J Immunol 173: 2562–2570. 15294972 10.4049/jimmunol.173.4.2562

[29] Nilsson A, Vesterlund L, Oldenborg PA (2012) Macrophage expression of LRP1, a receptor for apoptotic cells and unopsonized erythrocytes, can be regulated by glucocorticoids. Biochem Biophys Res Commun 417: 1304–1309. 22234309 10.1016/j.bbrc.2011.12.137

[30] Chao MP, Alizadeh AA, Tang C, Myklebust JH, Varghese B, et al (2010) Anti-CD47 antibody synergizes with rituximab to promote phagocytosis and eradicate non-Hodgkin lymphoma. Cell 142: 699–713. 20813259 10.1016/j.cell.2010.07.044 PMC2943345

[31] Willingham SB, Volkmer JP, Gentles AJ, Sahoo D, Dalerba P, et al (2012) The CD47-signal regulatory protein alpha (SIRPa) interaction is a therapeutic target for human solid tumors. Proc Natl Acad Sci U S A 109: 6662–6667. 22451913 10.1073/pnas.1121623109 PMC3340046

